# Splenomegaly in predicting the survival of patients with advanced primary liver cancer treated with immune checkpoint inhibitors

**DOI:** 10.1002/cam4.4818

**Published:** 2022-05-23

**Authors:** Lu‐Shan Xiao, Cheng‐Yi Hu, Hao Cui, Rui‐Ning Li, Chang Hong, Qi‐Mei Li, Chao‐Yi Huang, Zhong‐Yi Dong, Hong‐Bo Zhu, Li Liu

**Affiliations:** ^1^ Big Data Center, Nanfang Hospital Southern Medical University Guangzhou China; ^2^ Department of Infectious Diseases, Nanfang Hospital Southern Medical University Guangzhou China; ^3^ Department of Infectious Diseases, Guangzhou First People's Hospital School of Medicine, South China University of Technology Guangzhou China; ^4^ Department of Radiation Oncology, Nanfang Hospital Southern Medical University Guangzhou China; ^5^ Department of Oncology, The First Affiliated Hospital, Hengyang Medical School University of South China Hengyang China

**Keywords:** immune checkpoint inhibitors, immune therapy, primary liver cancer, prognosis, splenomegaly

## Abstract

**Background & aims:**

Immune checkpoint inhibitors (ICIs) play an increasingly important role in the treatment of primary liver cancer (PLC). Some patients with PLC experience symptoms of splenomegaly. Splenomegaly may affect the efficacy of ICIs due to an imbalance of the immune microenvironment. Currently, there is a lack of evidence on the relationship between splenomegaly and prognosis in patients with PLC treated with ICIs. This study analyzed the relationship between splenomegaly and prognosis in patients with PLC treated with ICIs.

**Methods:**

In this retrospective cohort study of 161 patients with PLC treated with ICIs, splenomegaly was diagnosed using computed tomography or magnetic resonance imaging and the impact of splenomegaly on patient survival was analyzed.

**Results:**

Through univariate and multivariate Cox regression analyses, we determined that splenomegaly was associated with shortened overall survival (*p* = 0.002) and progression‐free survival (*p* = 0.013) in patients with PLC treated with ICIs. Kaplan–Meier analysis further validated our results. The overall survival and progression‐free survival of patients with splenomegaly were significantly shorter than those of patients without splenomegaly (*p* < 0.01 and *p* = 0.02, respectively).

**Conclusions:**

We concluded that splenomegaly was a predictor of prognosis in patients with PLC treated with ICIs. This is the first study to report this important finding.

## Graphical abstract

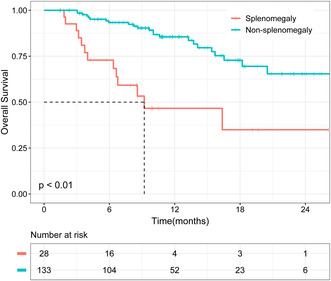

## 1 INTRODUCTION

Primary liver cancer (PLC) is the sixth most common cancer worldwide and the second most common cause of cancer‐related deaths.^1^ The incidence of PLC is increasing worldwide.^2^ By 2030, the number of patients with PLC is expected to be not <1.57 million, with an age‐standardized incidence rate of 14.08 per 100,000.^3^ Between 2012 and 2017, the age‐standardized death rate increased from 10.1 to 10.2 per 100,000.^4^

Immune checkpoint inhibitors (ICIs) play an increasingly important role in the treatment of PLC.^5^, ^6^ Immune checkpoint inhibitors are monoclonal antibodies that can block the binding of checkpoint proteins and their ligands on T cells to prevent T‐cell inactivation, thereby exerting antitumor effects.^7^, ^8^ According to reports, the combination of atezolizumab (an ICI) and bevacizumab significantly prolong overall survival (OS) and progression‐free survival (PFS) in patients with advanced hepatocellular carcinoma.^9^, ^10^ According to the American Society of Clinical Oncology guidelines,^11^ the combination of atezolizumab and bevacizumab is recommended as the first‐line treatment for advanced liver cancer. However, the objective response rates of ICI therapy were only 15–20%.^12^ Therefore, it is important to identify clinical characteristics that may predict the efficacy of ICIs, to screen the dominant population for ICI efficacy.

The spleen is the largest lymphoid organ in the human body and contains multiple immune cell subgroups.^13^, ^14^ According to previous reports, patients with splenomegaly may have splenic dysfunction and immune microenvironment disorders.^13^ The presence of splenomegaly in patients may affect the efficacy of ICIs due to an imbalance of the immune microenvironment. Therefore, we speculate that splenomegaly may affect the OS of patients with PLC treated with ICIs.

Thus far, there have been few clinical studies on liver cancer related to splenomegaly. Studies have reported that in patients with hepatitis B, splenomegaly is related to the incidence of hepatocellular carcinoma and OS.^15^ Splenomegaly may indicate hepatic decompensation after hepatectomy in patients with hepatocellular carcinoma and cirrhosis.^16^ Furthermore, among patients with hepatocellular carcinoma and portal vein tumor thrombus, patients with splenomegaly have worse OS.^17^ To date, no studies have reported the relationship between splenomegaly and prognosis, including OS and PFS in patients with PLC treated with ICIs. Therefore, this study aimed to objectively assess the relationship between splenomegaly and prognosis in patients with PLC treated with ICIs.

## 2 METHODS

### 2.1 Patient cohort and study design

**FIGURE 1:**
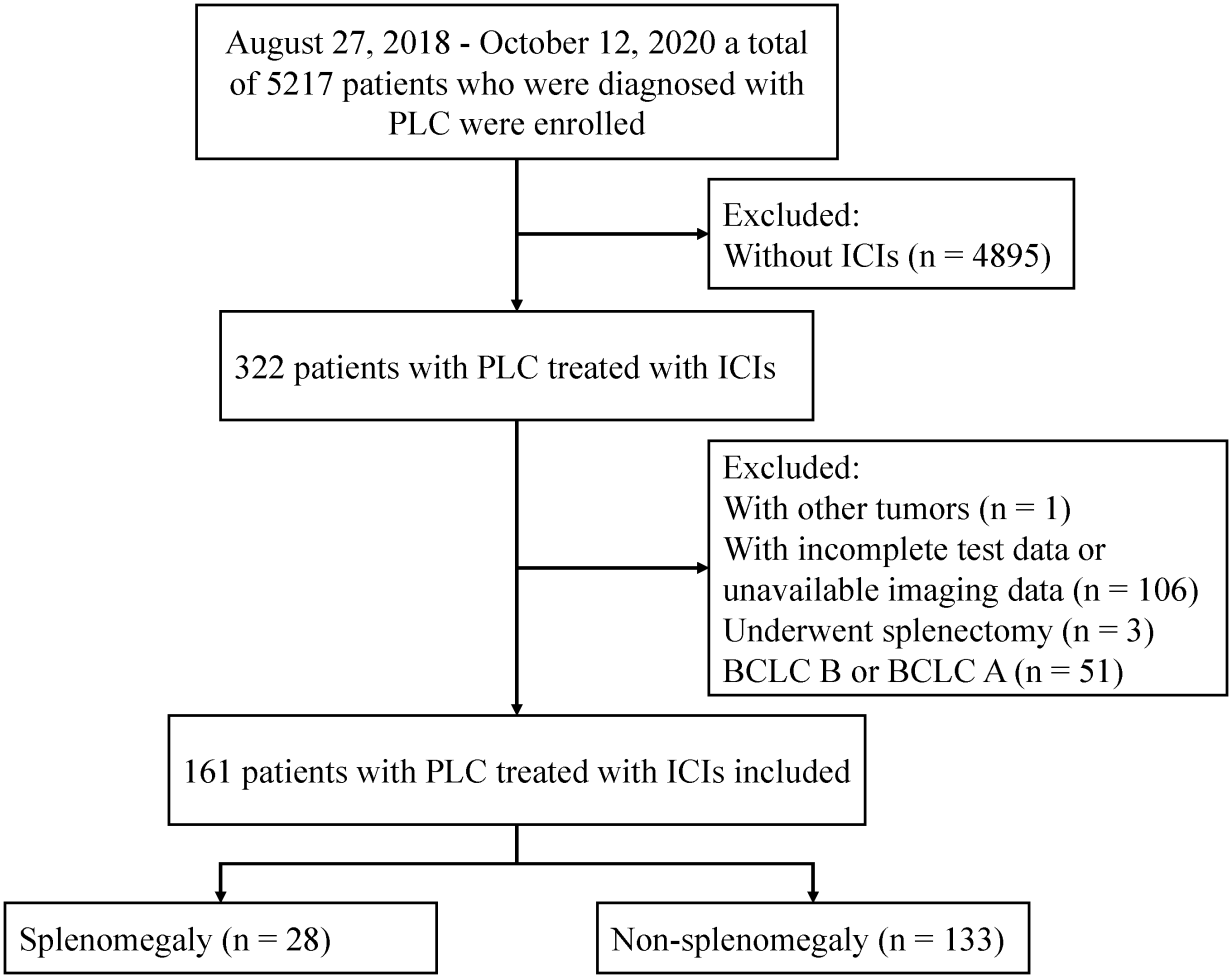
Flowchart of the patient selection process. *N*, number of patients

This was a single‐center retrospective study. Between August 27, 2018 and October 12, 2020, a total of 322 patients who were diagnosed with PLC and treated with ICIs were enrolled in the study. Figure 1 shows a flowchart of the patient selection process. The diagnosis of all patients with PLC met the diagnostic criteria of the Guidelines for the Diagnosis and Treatment of Hepatocellular Carcinoma (2019 edition).^18^ One patient with another type of tumor, 106 patients with incomplete test data or unavailable imaging data, three patients who underwent splenectomy, and 51 patients with Barcelona Clinic Liver Cancer (BCLC) stage A or B hepatocellular carcinoma were excluded. Finally, 161 patients with PLC treated with ICIs were enrolled. Among them, 28 had symptoms of splenomegaly and 133 had no symptoms of splenomegaly. The endpoints of this study were death and tumor progression. The baseline was defined as the start of ICI treatment. All the patients in our study (with and without splenomegaly) came back for follow‐up CT or MRI, which was performed according to their immunotherapy cycle. All procedures performed in studies involving human participants were in accordance with the ethical standards of the institutional and/or national research committee and with the 1964 Declaration of Helsinki and its later amendments or comparable ethical standards. The study design was approved by the Medical Ethics Committee of Nanfang Hospital, Southern Medical University (approval number: NFEC‐2021‐048). The requirement for written informed consent was waived owing to the retrospective nature of the study.

### 2.2 Definition of splenomegaly

The “gold standard” definition of splenomegaly is based on the spherical weight^19^; however, it is difficult to evaluate the weight of the spleen clinically. Because computed tomography and magnetic resonance imaging are simple and accurate methods for assessing whether patients have splenomegaly,^20^, ^21^ we chose to use computed tomography or magnetic resonance imaging to determine whether patients had splenomegaly. When the maximum diameter of the spleen was >15 cm, the patient was diagnosed with splenomegaly.^22^

### 2.3 Data collection

Baseline data, including information on age, sex, body mass index (BMI), history of smoking and drinking, BCLC stage, Child–Pugh class, Eastern Cooperative Oncology Group performance status (ECOG‐PS), portal vein tumor thrombus (PVTT), comorbidities, treatment before and after receiving ICIs, and laboratory examinations, were collected from the Electronic Medical Record System at the start of ICI treatment for each patient enrolled. PFS was defined as the time from treatment initiation to progression according to the Response Evaluation Criteria in Solid Tumors (version 1.1). OS was defined as the time from treatment initiation to death or last follow‐up. PFS was determined based on a review of electronic medical records, and OS was determined by telephone follow‐up. A team of well‐trained doctors and researchers collaborated to cross‐check patient data and verify the completeness and accuracy of the data. The entry time was defined as the time to the start of immunotherapy, and the exit time was defined as the time of death or last follow‐up.

### 2.4 Statistical analyses

Due to the small sample size of this study, the Shapiro–Wilk normality test was used to assess the normality of the data. Continuous variables with normal distribution were expressed as mean ± standard deviation, continuous variables with non‐normal distribution were expressed as median and interquartile range, and categorical variables were expressed as frequency and percentage. For continuous variables with normal distribution and non‐normal distribution, the *t*‐test and Mann–Whitney *U* test were used to compare the differences between groups, respectively. For categorical variables, the chi‐squared test or Fisher's exact test was used as appropriate. For the ranked variables, the rank‐sum test was used. Multivariate Cox regression analysis was used to further screen for influencing factors. Kaplan–Meier analysis was used to compare the survival rates, and the log‐rank test was used to compare the differences in survival curves between the groups. All statistical analyses were conducted using SPSS (version 25.0; IBM Corp., Armonk, NY, USA). All tests were two‐sided, and factors with *p* ≤ 0.05 were considered statistically significant.

## 3 RESULTS

### 3.1 Baseline characteristics of patients with PLC treated with ICIs

The baseline characteristics of the patients are shown in Table 1. This study included 161 patients with PLC who were treated with ICIs. More than 22% of patients were >60 years of age, more than 89% of patients were men, more than 36% of patients had a history of drinking, and more than 43% of patients had a history of smoking. The average BMI of all patients was 22.71 kg/m^2^. The ECOG‐PS of most patients was 0–1, and nearly 80% of patients were in Child–Pugh class A. No patient had an ECOG‐PS of 3, and no patient was in Child–Pugh class C. Cirrhosis (74.53%), and hepatitis B (90.06%) were very common comorbidities. Moreover, approximately 17% of patients had splenomegaly. More than 54% of the patients had PVTT. More than 70% of the patients with splenomegaly had PVTT, while only more than 50% of the patients without splenomegaly had PVTT. However, there was no significant difference between them (Figure S1). The treatment options for patients before receiving ICIs included surgery (23.60%), ablation (21.74%), transcatheter arterial chemoembolization (56.52%), and hepatic arterial infusion chemotherapy (14.29%). White blood cell counts, neutrophil counts, aspartate transaminase levels, alanine transaminase levels, and prothrombin times in most patients were within the normal range. Approximately one third of patients developed lymphopenia and hypoalbuminemia.

**TABLE 1:** The baseline characteristics of all patients

Characteristics	All patients (*n* = 161)
Age (≥60 years)	36 (22.36)
Male	144 (89.44)
BMI	22.71 ± 3.46
Drinking history	58 (36.02)
Smoking history	70 (43.48)
ECOG‐PS	
0	81 (50.31)
1	67 (41.61)
2	13 (8.08)
The lines of therapy	
First‐line therapy	130 (80.75)
Second‐line therapy	31 (19.25)
Child–Pugh Class	
A	128 (79.50)
B	33 (20.50)
Ascites	33 (20.50)
PVTT	88 (54.66)
Comorbidity	
Hypertension	32 (19.88)
Diabetes	18 (11.18)
Hepatitis B	145 (90.06)
Liver cirrhosis	120 (74.53)
Splenomegaly	28 (17.39)
Previous treatment	
Surgery	38 (23.60)
Ablation	35 (21.74)
TACE	91 (56.52)
HAIC	23 (14.29)
Laboratory finding	
WBC count (×10^9^/L)	5.80 (4.58–7.53)
Lymphocyte count (≥1.1 × 10^9^/L)	110 (68.32)
Neutrophil count (×10^9^/L)	3.57 (2.74–4.99)
AST (U/L)	44 (30–66)
ALT (U/L)	34 (22–51)
Total bilirubin (≥34 μmol/L)	9 (5.59)
ALB (≥35 g/L)	110 (68.32)
PT (s)	11.90 (11.00–12.70)
AFP (≥200 ng/ml)	98 (60.87)
Platelet count (<100 × 10^9^/L)	23 (14.29)

*Note*: Normally distributed continuous variable data are presented as mean ± standard deviation. Non‐normally distributed continuous variable data are presented as median (interquartile ranges). Classified variable data are presented as *n* (%).

Abbreviations: AFP, alpha‐fetoprotein; ALT, alanine aminotransferase; AST, aspartate aminotransferase; BMI, body mass index; ECOG‐PS, Eastern Cooperative Oncology Group performance status; HAIC, hepatic arterial infusion chemotherapy; PT, prothrombin time; PVTT, portal vein tumor thrombus; TACE, transcatheter arterial chemoembolization; WBC, white blood cell.

### 3.2 Univariate analysis of OS and PFS in patients with PLC treated with ICIs

In the univariate analysis, there were no significant differences in OS according to age, BMI, drinking history, smoking history, ECOG‐PS, Child–Pugh class, and previous baseline treatments (Table 2). Univariate analysis revealed that female sex (*p* = 0.019), splenomegaly (*p* < 0.001), and several baseline laboratory findings may be potentially influencing factors on the OS of patients with PLC treated with ICIs.

**TABLE 2:** Results of multivariate Cox regression analysis of OS in patients with PLC treated with ICIs

Characteristics	Univariate cox			Multivariate cox		
	Hazard ratio	95% Cl	*p*‐value	Hazard radio	95% Cl	*p*‐value
Age (≥60 years)	1.005	0.472–2.141	0.990			
Female	0.369	0.160–0.851	**0.019**	0.284	0.111–0.728	**0.009**
BMI	0.905	0.815–1.004	0.060			
Drinking history	0.631	0.286–1.391	0.254			
Smoking history	0.741	0.375–1.463	0.387			
ECOG‐PS						
1	1.296	0.640–2.622	0.471			
2	2.249	0.807–6.263	0.121			
Second‐line therapy	1.127	0.493–2.575	0.777			
Child‐Pugh Class(B)	1.771	0.853–3.677	0.125			
Ascites	1.179	0.552–2.515	0.671			
Comorbidity						
Hypertension	1.011	0.460–2.223	0.978			
Diabetes	0.970	0.374–2.513	0.950			
Hepatitis B	1.436	0.440–4.689	0.551			
Liver cirrhosis	1.375	0.626–3.02	0.427			
Splenomegaly	4.357	2.188–8.677	**<0.001**	3.997	1.517–10.531	**0.005**
Previous treatment						
Surgery	0.501	0.195–1.289	0.152			
Ablation	0.736	0.321–1.684	0.467			
TACE	1.185	0.605–2.319	0.621			
HAIC	0.961	0.337–2.738	0.940			
Laboratory finding						
WBC count (×10^9^/L)	0.907	0.780–1.056	0.210			
Lymphocyte count (≥1.1 × 10^9^/L)	0.437	0.227–0.844	**0.014**	0.990	0.423–2.319	0.982
Neutrophil count (×10^9^/L)	0.967	0.816–1.145	0.696			
AST(U/L)	1.003	1.000–1.006	**0.038**	1.004	1.000–1.007	**0.040**
ALT(U/L)	1.002	0.998–1.006	0.278			
Total bilirubin (≥34 μmol/L)	0.935	0.224–3.895	0.926			
ALB (≥35 g/L)	0.504	0.259–0.981	**0.044**	0.734	0.337–1.598	0.435
PT (s)	1.232	1.047–1.449	**0.012**	0.944	0.760–1.172	0.599
AFP (≥200 ng/ml)	2.664	1.213–5.851	**0.015**	2.411	1.067–5.448	**0.034**
Platelet count (<100 × 10^9^/L)	2.504	1.204–5.209	**0.014**	1.803	0.597–5.443	0.296

Abbreviations: AFP, alpha‐fetoprotein; ALB, albumin; ALT, alanine aminotransferase; AST, aspartate aminotransferase; BMI, body mass index; ECOG‐PS, Eastern Cooperative Oncology Group performance status; HAIC, hepatic arterial infusion chemotherapy; PT, prothrombin time; TACE, transcatheter arterial chemoembolization; WBC, white blood cell. In univariate cox, bold values indicate variables that were included in the multivariate cox regression analysis. In multivariate cox, bold values indicate statistically significance.

As shown in Table 3, splenomegaly was associated with PFS (*p* = 0.017). There were no significant differences in age, sex, BMI, drinking history, smoking history, ECOG‐PS, Child–Pugh class, ascites, previous treatments, and baseline laboratory findings.

**TABLE 3:** Results of multivariate Cox regression analysis of PFS in patients with PLC treated with ICIs

Characteristics	Univariate cox			Multivariate cox		
	Hazard ratio	95% Cl	*p*‐value	Hazard radio	95% Cl	*p*‐value
Age (≥60 years)	0.694	0.459–1.049	**0.083**	0.695	0.458–1.054	0.087
Female	0.630	0.383–1.038	**0.070**	0.554	0.334–0.921	**0.023**
BMI	0.971	0.925–1.020	0.243			
Drinking history	1.295	0.911–1.840	0.149			
Smoking history	0.835	0.596–1.168	0.292			
ECOG‐PS						
1	0.928	0.654–1.316	0.674			
2	1.623	0.891–2.956	0.114			
Second‐line therapy	1.127	0.493–2.575	0.777			
Child–Pugh Class(B)	1.011	0.665–1.537	0.959			
Ascites	1.012	0.664–1.542	0.956			
Comorbidity						
Hypertension	0.868	0.574–1.313	0.503			
Diabetes	0.902	0.539–1.510	0.694			
Hepatitis B	1.571	0.902–2.739	0.111			
Liver cirrhosis	1.355	0.915–2.005	0.130			
Splenomegaly	1.708	1.101–2.650	**0.017**	1.766	1.130–2.761	**0.013**
Previous treatment						
Surgery	1.256	0.850–1.854	0.253			
Ablation	1.084	0.729–1.611	0.690			
TACE	1.311	0.937–1.836	0.114			
HAIC	1.347	0.860–2.111	0.194			
Laboratory finding						
WBC count (×10^9^/L)	0.998	0.926–1.076	0.960			
Lymphocyte count (≥1.1 × 10^9^/L)	0.788	0.554–1.121	0.185			
Neutrophil count (×10^9^/L)	1.022	0.939–1.113	0.611			
AST (U/L)	1.000	0.997–1.003	0.955			
ALT (U/L)	1.001	0.998–1.004	0.511			
Total bilirubin (≥34 μmol/L)	0.684	0.301–1.554	0.364			
ALB (≥35 g/L)	1.058	0.739–1.513	0.759			
PT (s)	1.025	0.919–1.144	0.654			
AFP (≥200 ng/ml)	1.329	0.941–1.875	0.106			
Platelet count (<100 × 10^9^/L)	1.207	0.997–1.001	0.439			

Abbreviations: AFP, alpha‐fetoprotein; ALB, albumin; ALT, alanine aminotransferase; AST, aspartate aminotransferase; BMI, body mass index; ECOG‐PS, Eastern Cooperative Oncology Group performance status; HAIC, hepatic arterial infusion chemotherapy; PT, prothrombin time; TACE, transcatheter arterial chemoembolization; WBC, white blood cell. In univariate cox, bold values indicate variables that were included in the multivariate cox regression analysis. In multivariate cox, bold values indicate statistically significance.

### 3.3 Multivariate cox regression analysis of OS and PFS in patients with PLC treated with ICIs

To further clarify the impact of splenomegaly on the OS and PFS of patients with PLC treated with ICIs, we included variables with *p* < 0.05 for OS and *p* < 0.1 for PFS in the univariate analysis in the multivariate Cox regression analysis. For OS, these included sex (*p* = 0.019), splenomegaly (*p* < 0.001), lymphocyte count (*p* = 0.014), aspartate transaminase (*p* = 0.038), albumin (*p* = 0.044), prothrombin time (*p* = 0.012), and alpha‐fetoprotein (AFP) (*p* = 0.015). For PFS, these included age (*p* = 0.083), sex (*p* = 0.070), and splenomegaly (*p* = 0.017). Multivariate Cox regression analysis revealed that sex (*p* = 0.015), splenomegaly (*p* = 0.002), and AFP (*p* = 0.035) were risk factors associated with OS in patients with PLC treated with ICIs, and that sex (*p* = 0.023) and splenomegaly (*p* = 0.013) were risk factors associated with PFS. Although the univariate analysis revealed that platelet count (*p* = 0.014) may be a potential factor for the OS of patients with PLC treated with ICIs, the multivariate Cox regression analysis revealed that platelet count was not a risk factor for OS.

### 3.4 Impact of splenomegaly on patients with PLC treated with ICIs

**FIGURE 2:**
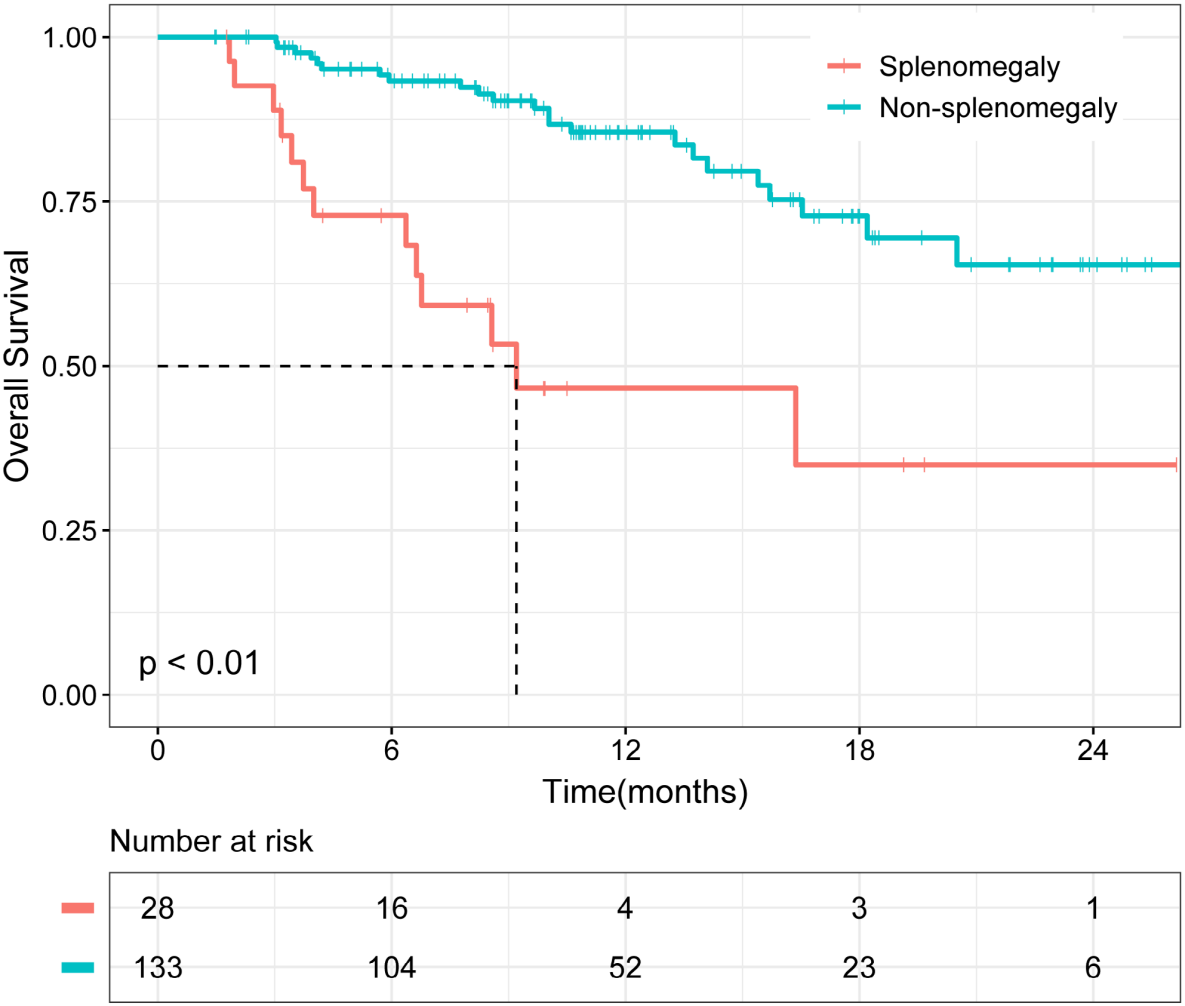
Kaplan–Meier analysis of overall survival (OS) in patients with splenomegaly and non‐splenomegaly.

**FIGURE 3:**
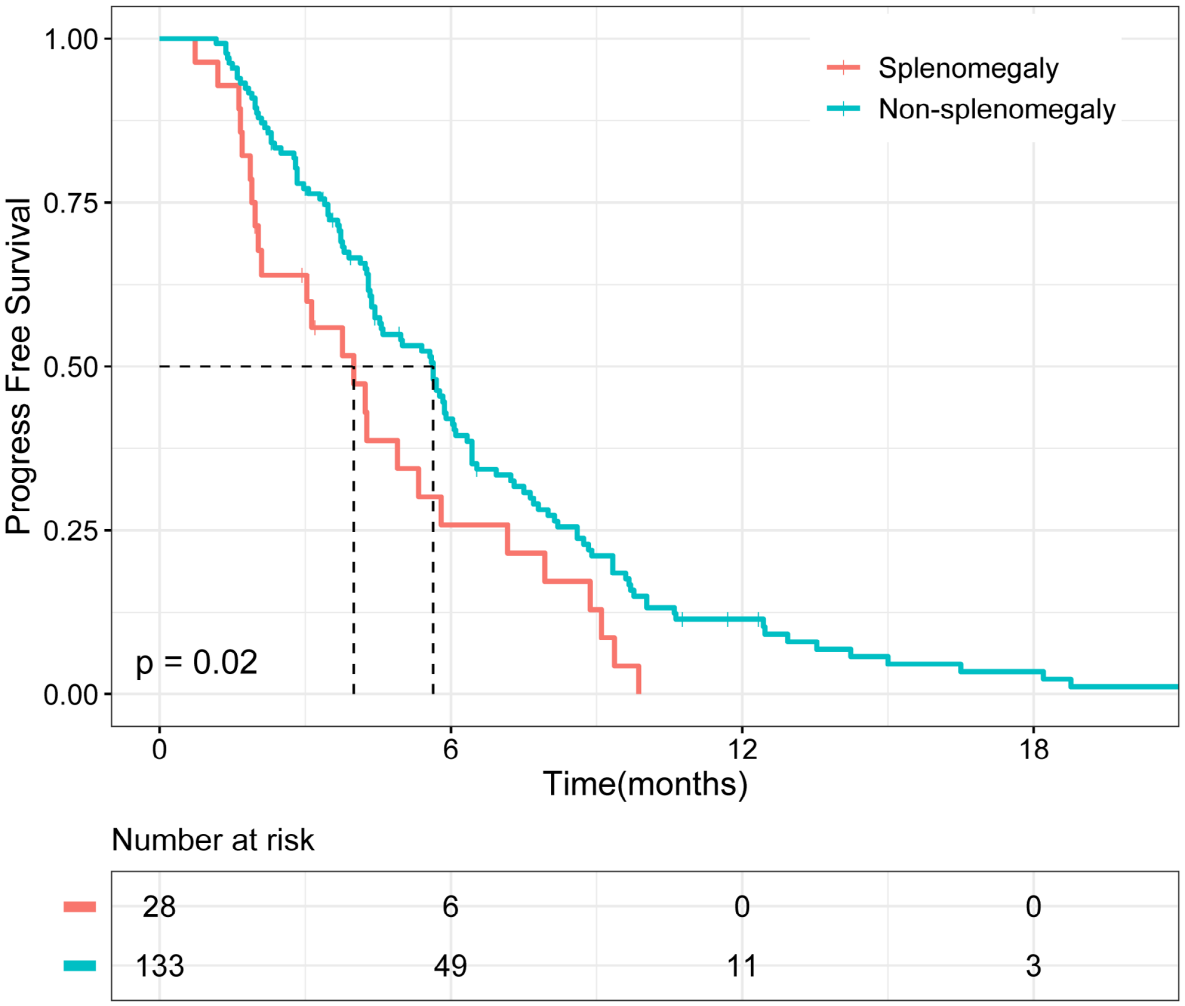
Kaplan–Meier analysis of progression‐free survival (PFS) in patients with splenomegaly and non‐splenomegaly.

To further verify the reliability of our results, we divided patients into a splenomegaly group (*n* = 28) and a non‐splenomegaly group (*n* = 133). As shown in Figure 2, Kaplan–Meier analysis demonstrated that patients with splenomegaly had worse OS than those without splenomegaly (*p* < 0.01). Similarly, PFS was worse in patients with splenomegaly (*p* = 0.02) (Figure 3).

As univariate analysis revealed that AFP and sex may be risk factors for OS in patients with PLC treated with ICIs, we conducted a stratified analysis based on AFP and sex. The results showed that splenomegaly was a significant factor affecting OS and PFS in patients with PLC treated with ICIs, regardless of sex (Figures S1 and S1). Among patients treated with ICIs with AFP levels ≥200 ng/ml, the OS of patients with splenomegaly was significantly lower than that of those without splenomegaly (*p* < 0.01). This trend was also observed in patients with AFP levels <200 ng/ml; however, there was no significant difference. Similarly, among patients with AFP levels ≥200 ng/ml, the PFS of patients with splenomegaly was significantly lower than that of those without splenomegaly (*p* = 0.04). However, we did not observe any significant differences among patients with AFP levels <200 ng/ml. In summary, splenomegaly could predict OS and PFS in patients with PLC treated with ICIs. Among patients with PLC treated with ICIs, those with splenomegaly had worse OS and PFS.

## 4 DISCUSSION

Our study is the first to report a predictor of OS and PFS in patients with PLC treated with ICIs. Among patients with PLC treated with ICIs, the survival probability of patients with splenomegaly was significantly lower than that of those without splenomegaly, which is of great clinical significance. The mechanism of poor prognosis in patients with PLC and splenomegaly is not clear. We speculate that splenomegaly can cause hemocytopenia, and anemia is associated with shorter survival times in patients with some types of cancer.^23^ However, the specific mechanism still needs to be explored by designing experiments. In this study, the incidence of splenomegaly was approximately 17% in patients with PLC treated with ICIs.

There are many causes of splenomegaly. In this study, splenomegaly was caused by obstruction of blood flow through the splenic vasculature and accumulation of blood in the red pulp due to liver cirrhosis.^14^ This may also be related to increased splenic angiogenesis and fibrogenesis.^24^ However, to date, no studies have reported the mechanism by which splenomegaly affects the efficacy of ICIs. We speculate that this may be because splenomegaly affects the number or function of lymphocytes in the spleen, or because splenomegaly oppresses the abdominal organs, leading to a worse prognosis.

As a predictor, the advantage of splenomegaly was that the imaging technique for measuring the size of the spleen was simple, accessible, non‐invasive, and inexpensive. In a previous study, high‐affinity neoantigens correlated with better OS in patients with hepatocellular carcinoma.^25^ However, neoantigens were analyzed by whole‐exome sequencing, which is expensive and not easily accessible clinically. Furthermore, the presence of programmed death ligand 1 has also been reported to be associated with the efficacy of ICIs.^26^ However, programmed death ligand 1 expression was determined by immunohistochemistry, which was invasive and not easily accessible clinically. In another study,^27^ immune‐related adverse events predicted the efficacy of ICIs. Immune‐related adverse events are not observed in all patients; however, the size of each patient's spleen can be measured by imaging.

Our study had some limitations. First, the sample size of patients with splenomegaly was relatively small and needed to be expanded to improve the reliability of the results. Second, few patients with cirrhosis were diagnosed pathologically. In other words, most patients with cirrhosis were diagnosed by imaging. This limits the reliability of the liver cirrhosis data. Third, this was a retrospective single‐center study. A multicenter prospective study needs to be conducted to further demonstrate our results. Finally, the lack of a control group of patients who did not receive immunotherapy further limits the power of our analysis.

## 5 CONCLUSIONS

Splenomegaly is a predictor of prognosis in patients with PLC treated with ICIs. Among these patients, the survival probability of patients with splenomegaly was significantly lower than that of those without splenomegaly. This suggests that splenomegaly is important in patients with PLC treated with ICIs. In clinical practice, timely alleviation of hypersplenism may improve the survival of patients with PLC treated with ICIs.

## CONFLICT OF INTEREST

The authors have no conflict of interest to declare.

## AUTHOR CONTRIBUTIONS

All authors contributed to the article and approved the submitted version. Conception/design: Lu‐Shan Xiao, Cheng‐Yi Hu, and Li Liu; Collection and/or assembly of data: Lu‐Shan Xiao, Cheng‐Yi Hu, Hao Cui, Rui‐Ning Li, Qi‐Mei Li, Chang Hong, Chao‐Yi Huang, and Li Liu; Data analysis and interpretation: Lu‐Shan Xiao, Cheng‐Yi Hu, Hao Cui, Zhong‐Yi Dong, and Hong‐Bo Zhu; Manuscript writing‐original draft: Lu‐Shan Xiao, Cheng‐Yi Hu, Rui‐Ning Li, and Li Liu; Final revision and approval of manuscript: All authors.

## FUNDING INFORMATION

This work was supported by the National Natural Science Foundation of China [grant numbers: 81773008 and 81972897] and the Clinical Research Startup Program of Southern Medical University by High‐Level University Construction Funding of Guangdong Provincial Department of Education [grant number: LC2019ZD003], the China Postdoctoral Science Foundation [grant number: 2021 M701629].

## ETHICS APPROVAL STATEMENT

All procedures performed in studies involving human participants were in accordance with the ethical standards of the institutional and/or national research committee and with the 1964 Declaration of Helsinki and its later amendments or comparable ethical standards. The study design was approved by the Medical Ethics Committee of Nanfang Hospital, Southern Medical University (approval number: NFEC‐2021‐048).

## PATIENT CONSENT STATEMENT

The requirement for written informed consent was waived owing to the retrospective nature of the study.

## Supporting information


Figure S1
Figure S2
Figure S3

## Data Availability

The data the support the findings of this study are available from the corresponding author upon reasonable request.
