# Supplementary material for: Splenomegaly in predicting the survival of patients with advanced primary liver cancer treated with immune checkpoint inhibitors
Source: Cancer Med. 2022 May 23;11(24):4880–8. doi: 10.1002/cam4.4818 (PMC9761067; doi:10.1002/cam4.4818)
Supplement: Supplementary file 1 — Figure S1 Figure S2 Figure S3 [file CAM4-11-4880-s001.docx]

**Supplementary S1**

**
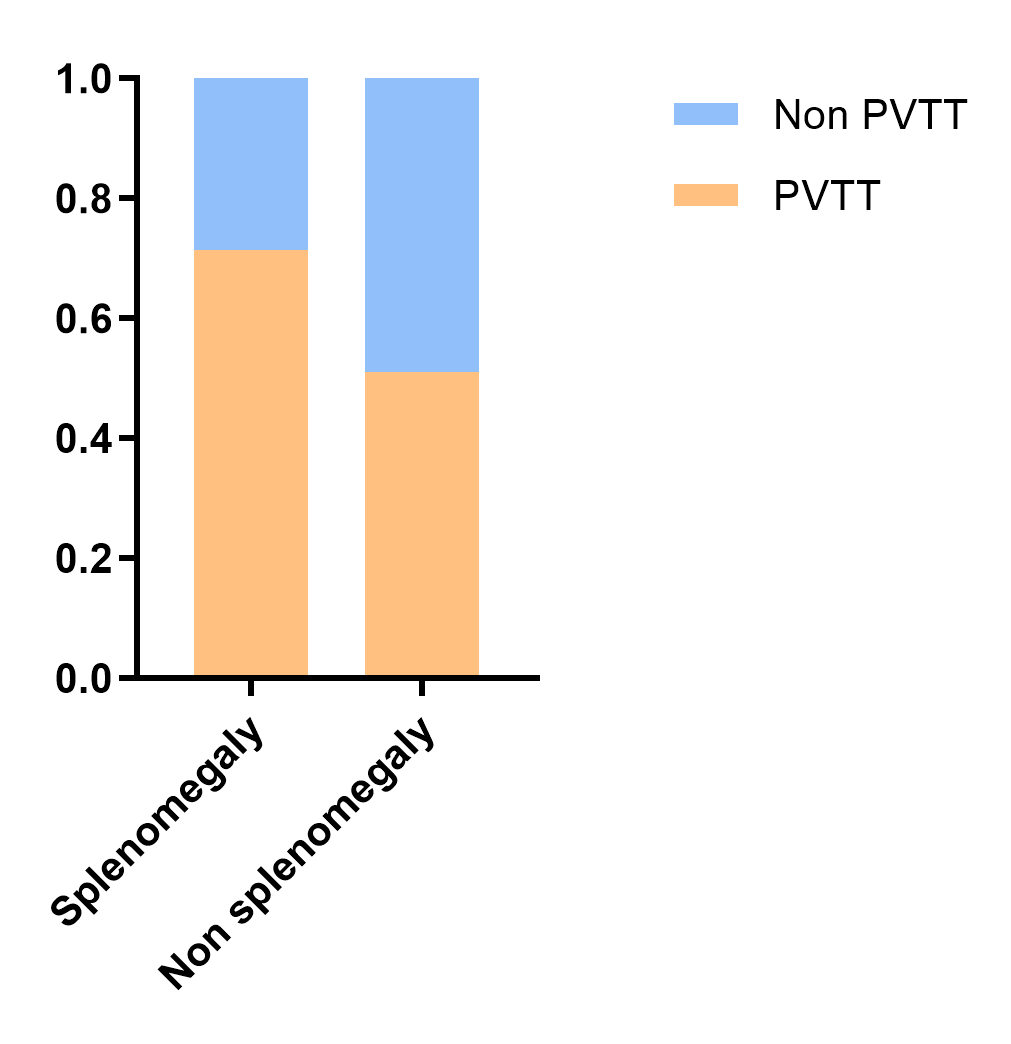
**

|  | Splenomegaly | Non splenomegaly | P-value |
| --- | --- | --- | --- |
|  |  |  | 0.050 |
| Non PVTT, n (%) | 8 (28.6) | 65 (48.9) |  |
| PVTT, n (%) | 20 (71.4) | 68 (51.1) |  |

Figure 1. The incidence of tumor thrombus in patients with splenomegaly and non splenomegaly. PVTT, portal vein tumor thrombus.

**Supplementary S2**


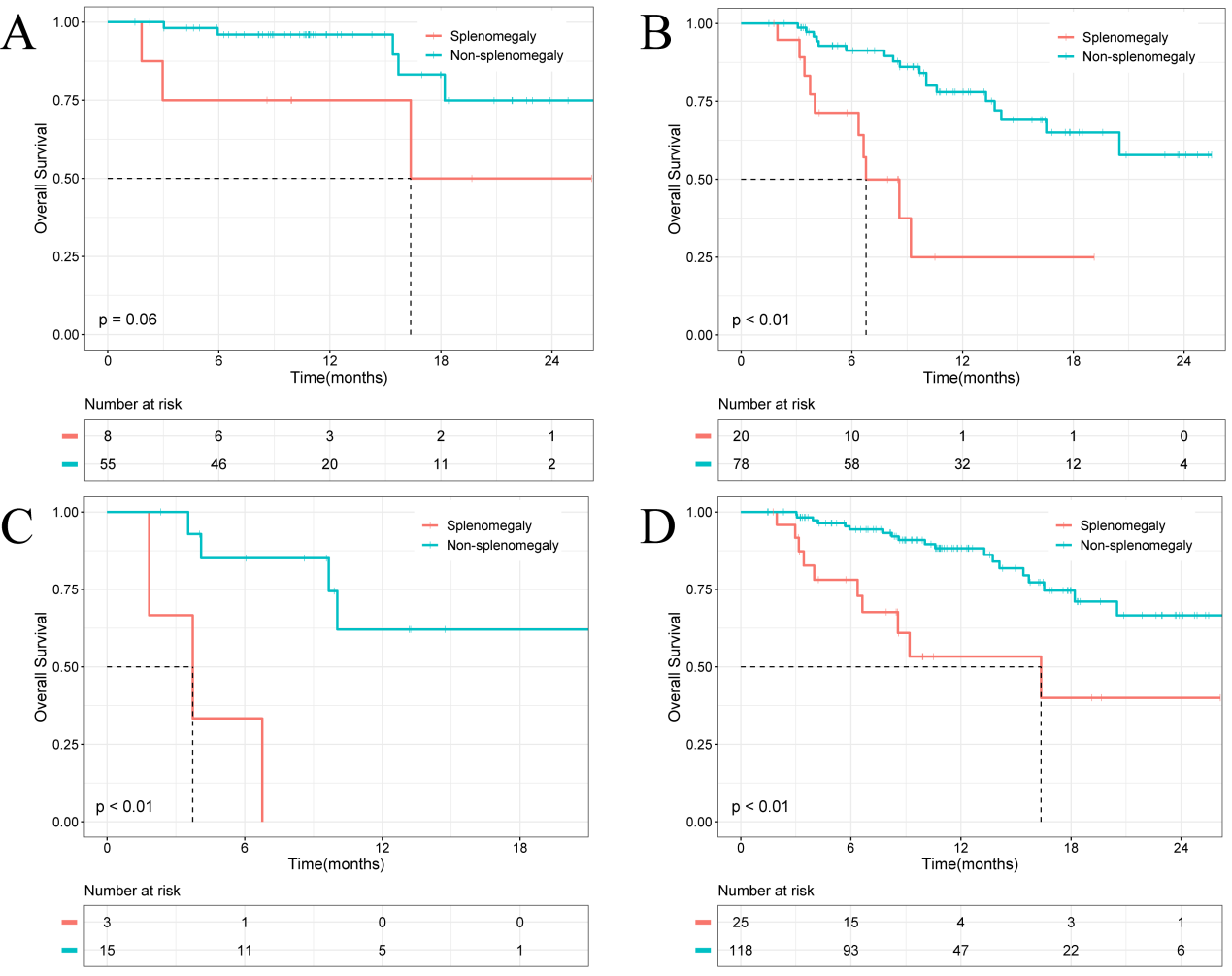


Kaplan-Meier survival analysis of OS in patients with AFP < 200ng/ml (A), patients with AFP ≥ 200ng/ml (B), females (C) and males (D).

**Supplementary S3**


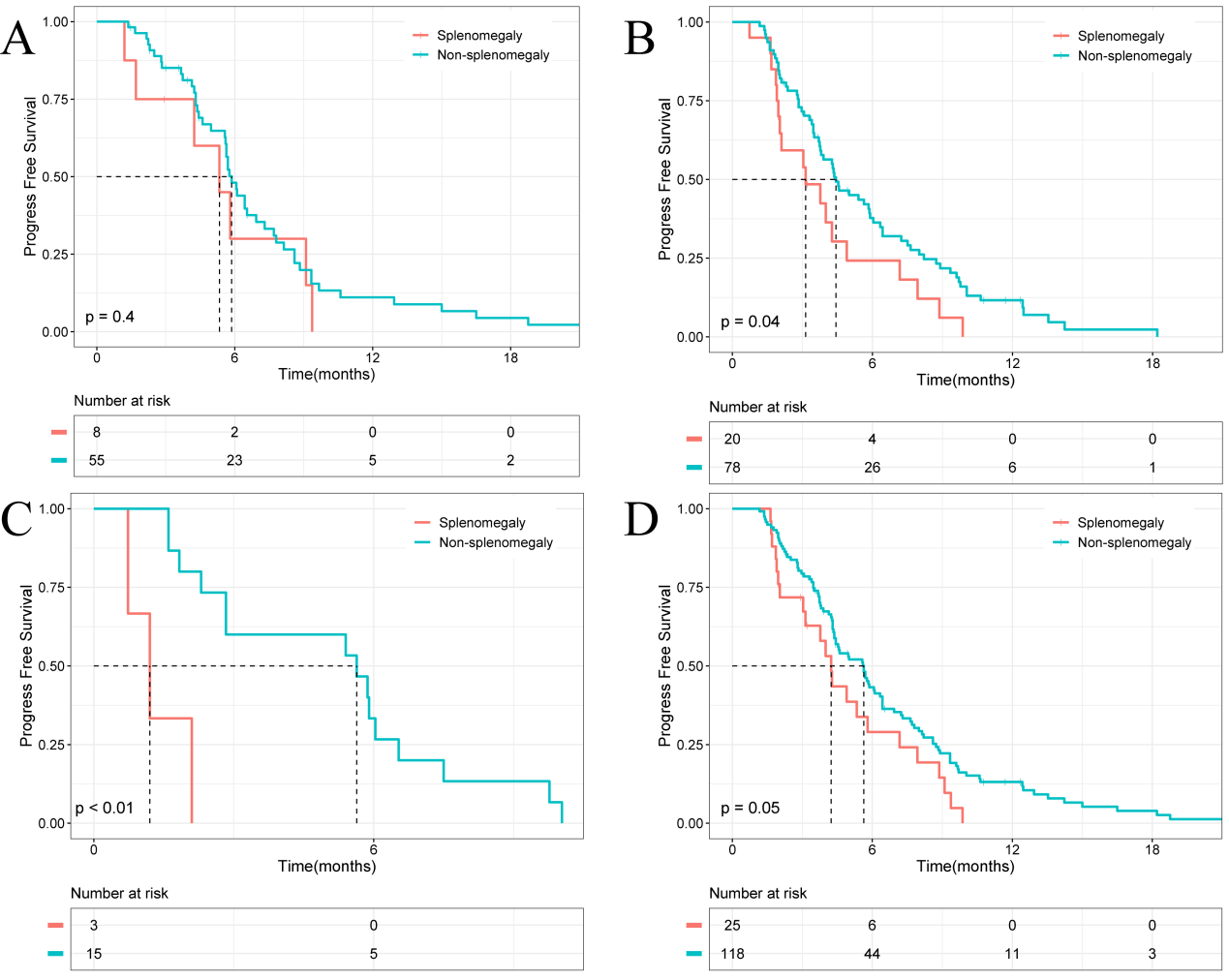


Kaplan-Meier survival analysis of PFS in patients with AFP < 200ng/ml (A), patients with AFP ≥ 200ng/ml (B), females (C) and males (D).
